# Supplementary material for: Nanosheet-Facilitated Spray Delivery of dsRNAs Represents a Potential Tool to Control Rhizoctonia solani Infection
Source: Int J Mol Sci. 2022 Oct 26;23(21):12922. doi: 10.3390/ijms232112922 (PMC9657606; doi:10.3390/ijms232112922)
Supplement: Supplementary file 1 [file ijms-23-12922-s001.zip › ijms-1961135-supplementary.pdf]

Supplemental materials

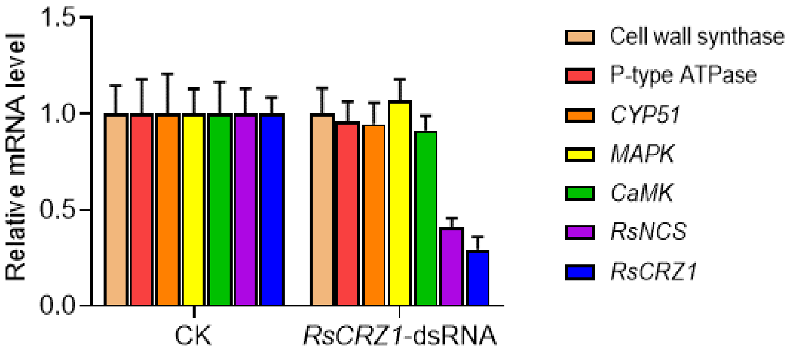

Figure S1. Expression levels of *Rs*CRZ1 and its downstream genes in *R. solani* after co-incubation with *Rs*CRZ1-dsRNA. RT-qPCR were performed to detect RNA levels for *Rs*CRZ1 and downstream genes, such as cell wall synthase gene, P-type ATPase gene, *CYP51*, *MAPK*, *CaMK* and *RsNCS*, in *R. solani* which was co-incubated with *Rs*CRZ1-dsRNA for 48 hours.

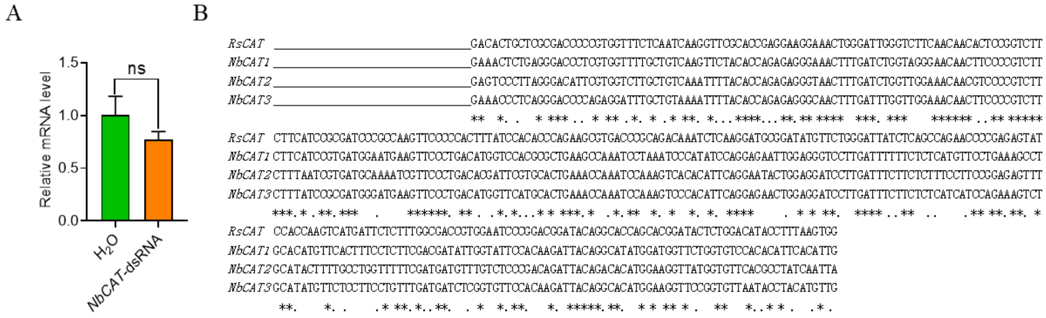

Figure S2. Expression levels of *Nb*CAT genes in tobacco sprayed with *Rs*CAT-dsRNA and their homologies with *Rs*CAT. (A) Expression levels of *NbCAT1*, *NbCAT2* and *NbCAT3* in tobacco 48 h after spraying with *Rs*CAT-dsRNA. ns, no significant differences (B) Sequences comparison of *Rs*CAT, *Nb*CAT1, *Nb*CAT2 and *Nb*CAT3. * indicates identical nucleotide.

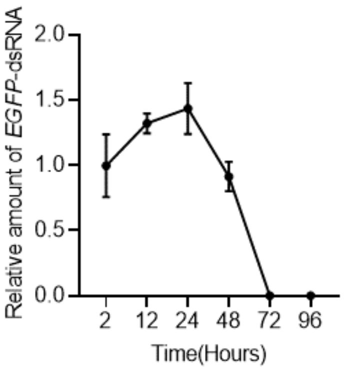

Figure S3. Detection of exogenous *EGFP*-dsRNA in the maize leaves at the different times after spray. *EGFP*-dsRNA in RNA samples collected at different time points was detected by RT-qPCR.

Table S1. Primers used to product dsRNA targeting different genes.

Name	Sequence (5′ →3′)
EGFP-dsF	cacatgaagcagcacgactt
EGFP-dsR	gttcaccttgatgccgttctt
EGFP-dsT7F	taatacgactcactatagggcacatgaagcagcacgactt
EGFP-dsT7R	taatacgactcactataggggttcaccttgatgccgttctt
RsCAT-dsF	gacactgctcgcgaccc
RsCAT-dsR	ccacttaaaggtatgtccagagt
RsCAT-dsT7F	taatacgactcactatagggggacactgctcgcgaccc
RsCAT-dsT7R	taatacgactcactatagggccacttaaaggtatgtccagagt
RsCRZ1-dsF	gctcgttctgtcggagatg
RsCRZ1-dsF	ctcatgtcgcttgcagtcat
RsCRZ1-T7dsF	taatacgactcactataggggctcgttctgtcggagatg
RsCRZ1-T7dsF	taatacgactcactatagggctcatgtcgcttgcagtcat
RsPG1-dsF	cgggaaagggtatcacattcaa
RsPG1-dsR	ggttcgagatgctgtaggtatg
RsPG1-dsT7F	taatacgactcactatagggcgggaaagggtatcacattcaa
RsPG1-dsT7R	taatacgactcactatagggggttcgagatgctgtaggtatg

Table S2. Primers used to qRT-PCR detection.

q*EGFP*-R	ccgaccactaccagcagaacac
q*EGFP*-R	tcacgaactccagcaggaccat
q*RsCAT*-F	ccacattgacctcctcgctcac
q*RsCAT*-R	gatggcagcacggcagatgtt
q*RsCRZ1*-F	tctcggctcaaacgggtctctc
q*RsCRZ1*-R	ttccgctccctgttcgctcat
q*RsGAPDH*-F	tgttgttggcgtgaacttggattc
q*RsGAPDH*-R	gcaccgtagtcatcaaaccctca
q*RsNCS*-F	cgaggaagccgaatacgagagc
q*RsNCS*-R	actgcatcgagcgaacagagga
q*RsPG1*F	ggatggtcagggcggtaatgga
q*RsPG1*R	tgtaggtatgtgcgggcgagtt
q*ZmUBQ9*-F	tgcggtgcgggtgtcttcat
q*ZmUBQ9*-R	tgcttgataggtaggcgggtga
